# Structural evidence for the partially oxidized dipyrromethene and dipyrromethanone forms of the cofactor of porphobilinogen deaminase: structures of the *Bacillus megaterium* enzyme at near-atomic resolution

**DOI:** 10.1107/S139900471303294X

**Published:** 2014-02-15

**Authors:** N. Azim, E. Deery, M. J. Warren, B. A. A. Wolfenden, P. Erskine, J. B. Cooper, A. Coker, S. P. Wood, M. Akhtar

**Affiliations:** a School of Biological Sciences, University of Punjab, New Campus, Lahore-54590, Pakistan; b School of Biosciences, University of Kent, Stacey Building, Canterbury CT2 7NJ, England; c Laboratory of Protein Crystallography, Centre for Amyloidosis and Acute Phase Proteins, UCL Division of Medicine (Royal Free Campus), Rowland Hill Street, London NW3 2PF, England

**Keywords:** tetrapyrrole biosynthesis, porphobilinogen deaminase, dipyrromethane cofactor

## Abstract

The enzyme porphobilinogen deaminase (PBGD; hydroxymethylbilane synthase; EC 2.5.1.61) catalyses an early step of the tetrapyrrole-biosynthesis pathway in which four molecules of the monopyrrole porphobilinogen are condensed to form a linear tetrapyrrole. The enzyme possesses a dipyrromethane cofactor, which is covalently linked by a thioether bridge to an invariant cysteine residue (Cys241 in the *Bacillus megaterium* enzyme). The cofactor is extended during the reaction by the sequential addition of the four substrate molecules, which are released as a linear tetrapyrrole product. Expression in *Escherichia coli* of a His-tagged form of *B. megaterium* PBGD has permitted the X-ray analysis of the enzyme from this species at high resolution, showing that the cofactor becomes progressively oxidized to the dipyrromethene and dipyrromethanone forms. In previously solved PBGD structures, the oxidized cofactor is in the dipyromethenone form, in which both pyrrole rings are approximately coplanar. In contrast, the oxidized cofactor in the *B. megaterium* enzyme appears to be in the dipyrromethanone form, in which the C atom at the bridging α-position of the outer pyrrole ring is very clearly in a tetrahedral configuration. It is suggested that the pink colour of the freshly purified protein is owing to the presence of the dipyrromethene form of the cofactor which, in the structure reported here, adopts the same conformation as the fully reduced dipyrromethane form.

## Abstract 2

The enzyme porphobilinogen deaminase (PBGD; hydroxymethylbilane synthase; EC 2.5.1.61) catalyses a key early step in the biosynthesis of tetrapyrroles in which four molecules of the monopyrrole porphobilinogen are condensed to form a linear tetrapyrrole. Two near-atomic resolution structures of PBGD from *B. megaterium* are reported that demonstrate the time-dependent accumulation of partially oxidized forms of the cofactor, including one that possesses a tetrahedral C atom in the terminal pyrrole ring.

## 1. Introduction   

**Figure 1:**
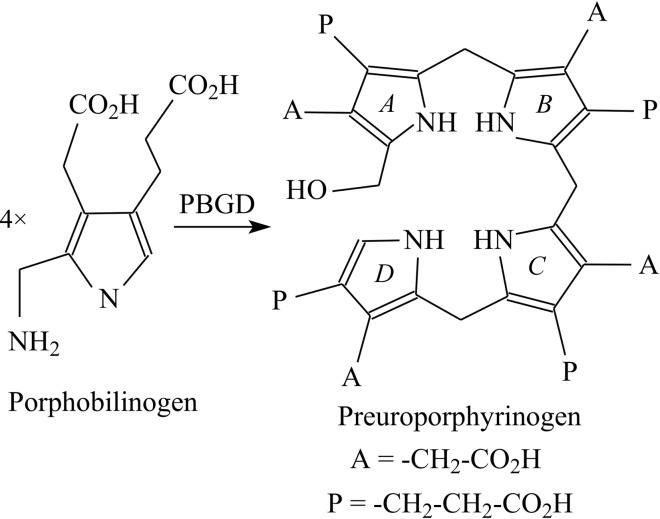
The reaction catalysed by porphobilinogen deaminase. Four molecules of the pyrrole porphobilinogen are condensed to form the linear tetrapyrrole preuroporphyrinogen (hydroxymethylbilane). The acetic acid and propionic acid side chains of each pyrrole are abbreviated A and P, respectively, and the four rings of the tetrapyrrole product are indicated in italics as *A*, *B*, *C* and *D*.

**Figure 2:**
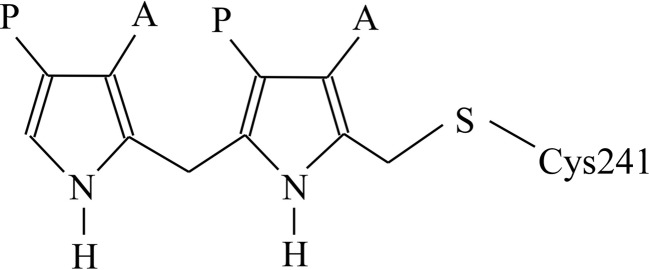
The dipyrromethane cofactor of porphobilinogen deaminase is covalently attached to the enzyme by a thioether bond to a cysteine residue. Four substrate pyrroles are added linearly to the cofactor; finally, hydrolysis of the linkage between the substrate and the cofactor releases the tetrapyrrole product hydroxymethylbilane.

Tetrapyrroles such as haem and chlorophyll play vital physiological roles in respiration and photosynthesis (Warren & Smith, 2009 ▶). One of the early steps in the biosynthesis of tetrapyrroles is catalysed by the enzyme porphobilinogen deaminase (PBGD), which is also known as hydroxy­methylbilane synthase (EC 2.5.1.61). This enzyme catalyses the polymerization of four molecules of the monopyrrole porphobilinogen in a stepwise head-to-tail manner to form the linear tetrapyrrole preuroporphyrinogen or hydroxymethyl­bilane (Fig. 1 ▶; Jordan, 1991 ▶). PBGDs are monomeric enzymes with molecular masses in the range 34–44 kDa depending on the species. The enzymes in this family exhibit high thermal stabilities and have pH optima in the range 8.0–8.5, with isoelectric points between 4.0 and 4.5. It has been shown that ring *A* of the tetrapyrrole product (Fig. 1 ▶) is the first to bind to the enzyme, followed by rings *B*, *C* and finally *D* (Jordan, 1991 ▶). The enzyme possesses a dipyrromethane cofactor (Fig. 2 ▶) that is covalently bound to the enzyme by a thioether linkage involving an invariant cysteine residue (Cys241 in the *Bacillus megaterium* enzyme; Jordan & Warren, 1987 ▶; Warren & Jordan, 1988 ▶; Scott *et al.*, 1988 ▶). Whilst the cofactor can be assembled slowly from two molecules of porphobilinogen, it can be generated more quickly by cleavage of the product, preuroporphyrinogen, which reacts rapidly with the apo­enzyme (Scott *et al.*, 1989 ▶; Awan *et al.*, 1997 ▶). During catalysis, the free α-position of the cofactor acts as the attachment point to which the growing tetrapyrrole chain is anchored. The four porphobilinogen substrate molecules (S) react sequentially with the enzyme (E) to generate stable ES_1_, ES_2_, ES_3_ and ES_4_ complexes. ES_4_ is therefore an enzyme-bound hexapyrrole, representing two pyrroles of the cofactor attached to the tetrapyrrole bilin product. After the assembly of ES_4_, cleavage of the link between the cofactor and the first substrate molecule completes the reaction, allowing the tetrapyrrole product to be released.

The X-ray structures of the *Escherichia coli*, human and *Arabidopsis* enzymes have been solved (Louie *et al.*, 1992 ▶, 1996 ▶; Hädener *et al.*, 1999 ▶; Gill *et al.*, 2009 ▶; Song *et al.*, 2009 ▶; Roberts *et al.*, 2013 ▶). The polypeptide is folded into three domains (1–3), each of approximately the same size. The topology of domains 1 and 2 shows a strong resemblance to the type II periplasmic binding proteins (Louie *et al.*, 1992 ▶; Louie, 1993 ▶), whereas domain 3 has an entirely distinct fold. The dipyrromethane cofactor is covalently attached to a cysteine residue in a loop of domain 3 so that it is positioned within a deep active-site cleft formed between domains 1 and 2. The active-site cleft is formed by several crucial arginine residues that bind the side-chain carboxylates of the cofactor and/or substrate. Indeed, many of the point mutations in the human PBGD gene which give rise to the disease acute intermittent porphyria affect these conserved arginine residues (Wood *et al.*, 1995 ▶).

*B. megaterium*, literally meaning ‘big beast’ (Vary, 1994 ▶), is a common Gram-positive soil bacterium that is highly versatile as its metabolism allows it to utilize inexpensive carbon sources and is capable of surviving in a divergent range of environments (Vary *et al.*, 2007 ▶). It has many commercial applications in food processing and in the biotechnological production of various drugs and vitamins, including the tetrapyrrole-derived vitamin B_12_ (Bunk *et al.*, 2010 ▶). The gene for PBGD in *B. megaterium* encodes a protein of 310 amino acids which has 48% sequence identity to the *E. coli* enzyme. Here, we report the structure analysis of PBGD from *B. megaterium* in a crystal form that diffracts synchrotron radiation to 1.5 Å resolution. The collection of data from two crystals, which were flash-cooled at periods of 40 and 50 d after purification, demonstrates that the dipyrromethane cofactor adopts two conformations corresponding to partially oxidized states. One of these is likely to be the dipyrromethene form, as it adopts essentially the same conformation as the fully reduced dipyrromethane. The other is likely to be the further oxidized dipyrromethanone form, which has not previously been observed structurally. This form possesses a tetrahedral ring-linking α-C atom in the terminal pyrrole, and the two structures presented here demonstrate that the proportion of the cofactor in this state increases as a function of time, with a concomitant decrease in the amount of the other conformer.

## 2. Methods   

The expression of *B. megaterium* PBGD in *E. coli* using a pET-­14b construct has been reported recently along with the purification and crystallization of the enzyme (Azim *et al.*, 2013 ▶). The affinity-purified enzyme was assayed spectrophotometrically using the method described in Shoolingin-Jordan *et al.* (1997 ▶). Since the dipyrromethane cofactor of PBGD is light-sensitive, crystals were grown in the dark using the hanging-drop method. Crystals of the enzyme could be obtained reproducibly in 0.1 *M* sodium cacodylate pH 6.5–6.8, 0.2 *M* magnesium acetate, 25–30% PEG 8K following removal of the polyhistidine tag and concentration of the enzyme to 2.5 mg ml^−1^. Selected crystals were treated by the addition of glycerol to approximately 30%(*v*/*v*) before mounting in loops and flash-cooling with an Oxford Cryosystems cryocooler. Data were collected from two crystals, one of which (crystal 1) was cooled approximately 40 d after purification of the protein and the other of which (crystal 2) was cooled when the protein was approximately 50 d old.

X-ray data collection on crystal 1 was undertaken at station I03 at the Diamond Light Source (DLS; Didcot, England) using a Pilatus 6M-F detector at a wavelength of 0.976 Å. Data were collected from crystal 2 on the European Synchrotron Radiation Facility (ESRF) beamline BM14 using a MAR Mosaic 225 CCD detector at a wavelength of 1.07 Å. Both data sets were collected at a temperature of 100 K using 1° oscillations and were processed with *MOSFLM* (Leslie, 2006 ▶; Powell *et al.*, 2013 ▶), *SCALA* (Evans, 2006 ▶) and other programs from the *CCP*4 suite (Winn *et al.*, 2011 ▶). Structure analysis was successful for the crystal 1 data set using the molecular-replacement program *MOLREP* (Vagin & Teplyakov, 2010 ▶) with *E. coli* PBGD (48% identity; PDB entry 1pda; Louie *et al.*, 1992 ▶) as the search model. Refinement and rebuilding of the *B. megaterium* PBGD structure was completed using this data set with the programs *REFMAC* (Murshudov *et al.*, 2011 ▶) and *Coot* (Emsley & Cowtan, 2004 ▶). Subsequently, the structure was refined as above using the crystal 2 data set, and further crystallographic details for both structures can be found in Table 1 ▶. The geometric restraints for refinement of the two forms of the cofactor were generated using *PRODRG* (Schüttelkopf & van Aalten, 2004 ▶). The final refined structures and reflection data sets were analysed by the validation programs *PROCHECK* (Laskowski *et al.*, 1993 ▶), *SFCHECK* (Vaguine *et al.*, 1999 ▶) and *RAMPAGE* (Lovell *et al.*, 2003 ▶; Chen *et al.*, 2010 ▶) and have been deposited in the Protein Data Bank (http://www.wwpdb.org) with accession codes 4mlv and 4mlq for crystals 1 and 2, respectively. Figures of the structures were prepared using *CueMol* (http://www.cuemol.org/en) and were rendered using *POV-Ray* (http://www.povray.org).

## 3. Results   

### 3.1. Structure analysis   

**Figure 3:**
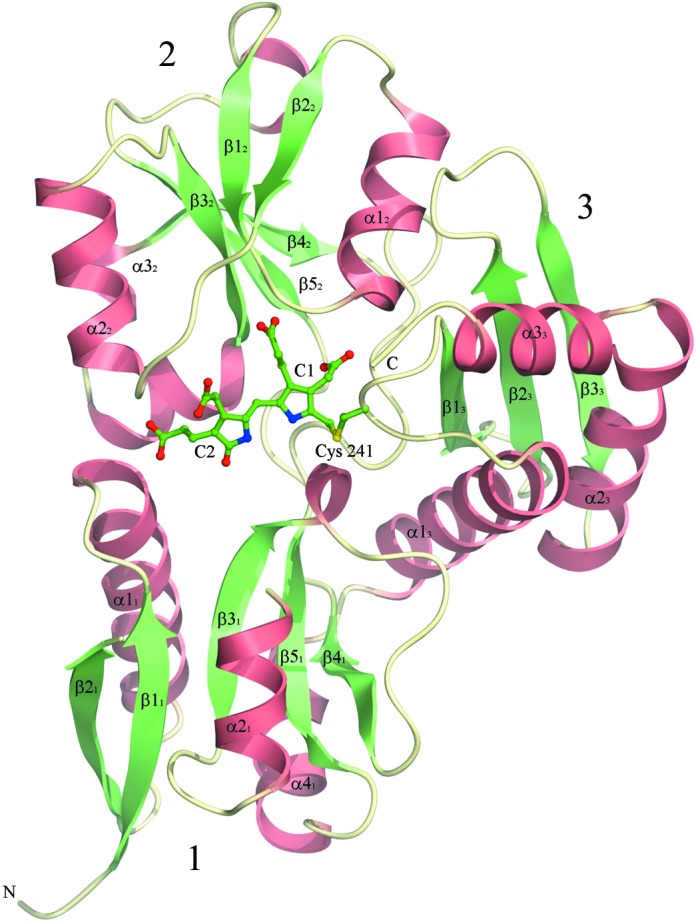
The tertiary structure of *B. megaterium* PBGD at 1.46 Å resolution showing the oxidized form of the dipyrromethane cofactor covalently attached to Cys241. The domains of the enzyme are numbered 1–3 and the secondary-structure elements are labelled according to the nomenclature of Louie *et al.* (1992 ▶).

The structure of *B. megaterium* PBGD has been determined at a resolution of 1.46 Å, with an *R* factor of 14.1% and an *R*_free_ of 18.6% (see Table 1 ▶). The resulting model of the enzyme was found to have 99% of the amino acids within the favoured regions of the Ramachandran plot and the remaining 1% within allowed areas according to the *RAMPAGE* criteria (Lovell *et al.*, 2003 ▶). The estimated r.m.s. coordinate error (Read, 1986 ▶) of 0.11 Å suggests that the structure is defined with high accuracy. In addition, the structure of the enzyme in which the cofactor has undergone more extensive oxidation is presented at the slightly lower resolution of 1.6 Å with similar refinement and validation statistics (see Table 1 ▶). The overall fold of the enzyme is shown in Fig. 3 ▶, in which the secondary-structure elements are labelled according to the notation of Louie *et al.* (1992 ▶).

**Figure 4:**
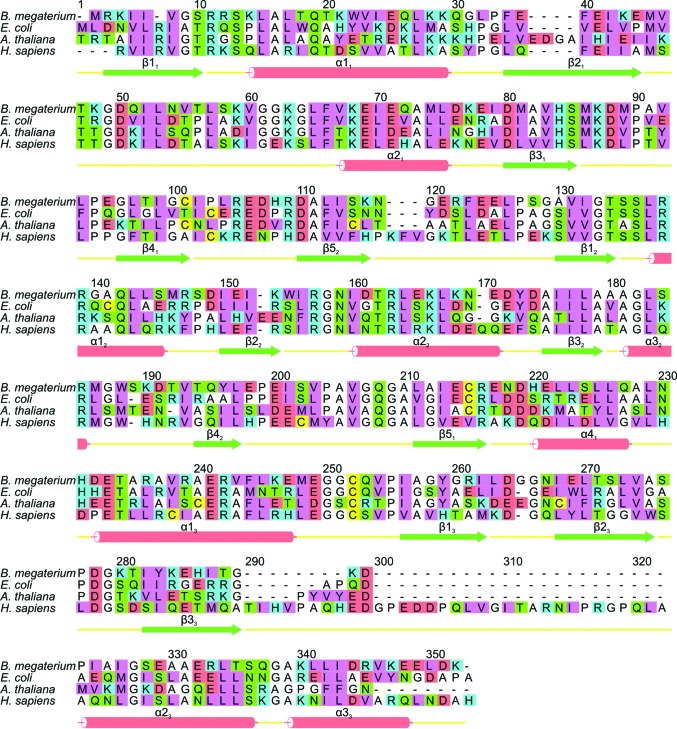
Sequence alignment and secondary structure of *B. megaterium* PBGD. An alignment of *B. megaterium* PBGD with the enzyme from another prokaryote (*E. coli*) along with the plant (*A. thaliana*) and human enzymes. The secondary-structure elements are labelled using the notation of Louie *et al.* (1992 ▶) and the amino-acid residues are colour-coded as follows: cyan, basic; red, acidic; green, neutral polar; pink, bulky hydrophobic; white, Gly, Ala and Pro; yellow, Cys.

All enzymes in this family adopt a three-domain fold in which domains 1 and 2 resemble the fold of type II periplasmic binding proteins and the third domain, to which the cofactor is covalently attached, adopts a distinct α+β topology. Starting at the N-terminal end, the backbone of *B. megaterium* PBGD follows that of the *E. coli* enzyme until the end of stand β2_1_, where there is a substantial disordered region extending from residues 42 to 62. This disordered region is known to form a flexible loop covering the active site and has only been defined structurally in the *Arabidopsis* enzyme (Roberts *et al.*, 2013 ▶). The ordered region of the *B. megaterium* structure resumes at residue 63, which is at the N-terminal end of helix α2_1_, and continues to follow the fold of the *E. coli* enzyme closely, with the exception of a few single-residue insertions in loop regions that are distant from the active site. The sequence alignment shown in Fig. 4 ▶ shows the marked similarity of enzymes in this family. Not surprisingly, the eukaryotic enzymes exhibit some significant differences from the prokaryotic PBGDs, *e.g.* the human enzyme has a large insertion towards the C-terminal end and the *Arabidopsis* enzyme appears to be somewhat truncated relative to the others at the same end of the molecule.

**Figure 5:**
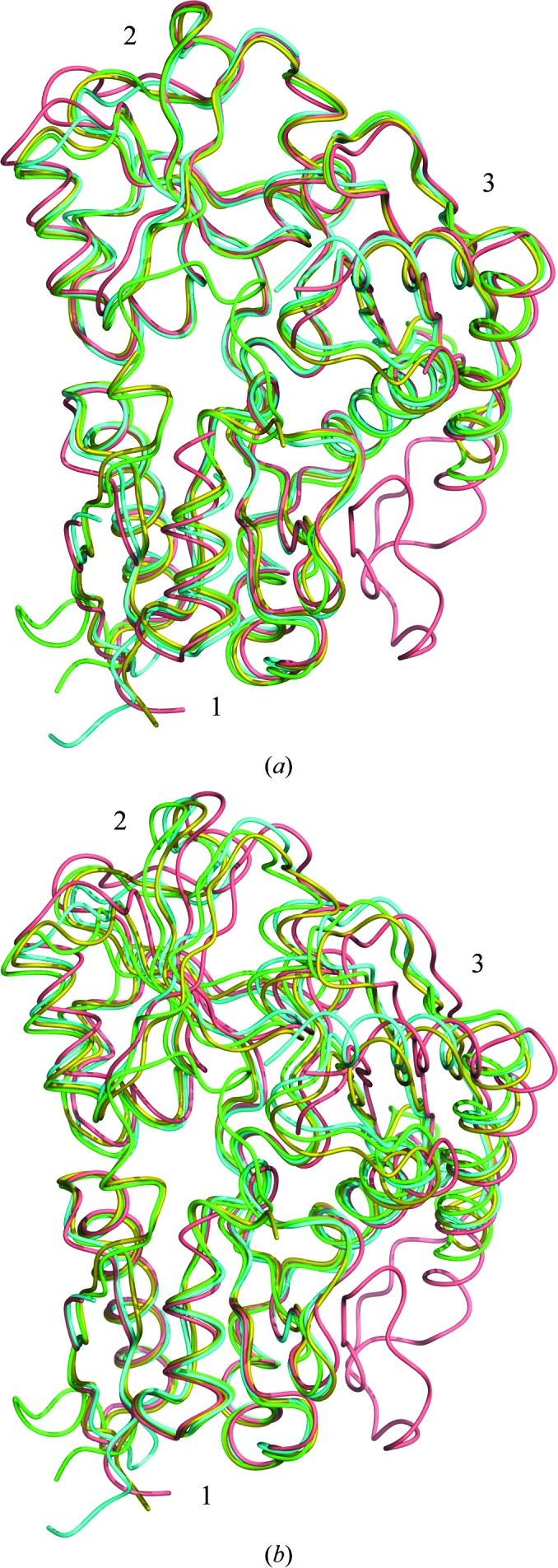
Superposition of *B. megaterium* PBGD with the *Arabidopsis*, *E. coli* and human enzymes. (*a*) The overall least-squares superposition of *B. megaterium* PBGD (cyan) with the *E. coli* enzyme (yellow) as well as the *Arabidopsis* and human enzymes (green and pink, respectively). A superposition of the four enzymes based on domain 1 alone is shown in (*b*), which emphasizes the different concerted shifts of domains 2 and 3 relative to domain 1 in the enzyme from each species.

The refined structure of *B. megaterium* PBGD superimposes with the *E. coli* and *Arabidopsis* enzymes (which are of comparable resolution) with r.m.s. C^α^ deviations of 1.05 and 1.02 Å for 280 and 271 structurally equivalent residues, respectively. Likewise, superposition with the structure of the human enzyme demonstrates a similarly good r.m.s.d. of 1.13 Å for 276 structurally equivalent C^α^ atoms. It has been suggested that the domains of this enzyme may move independently to allow substantial rearrangements of the polypyrrole during the elongation cycle (Louie *et al.*, 1992 ▶, 1996 ▶). Inspection of the *A. thaliana* PBGD structure suggested that domains 2 and 3 of the enzyme appear to move as a relatively rigid unit with respect to domain 1 (Roberts *et al.*, 2013 ▶). Indeed, the superpositions of *B. megaterium* PBGD with the *E. coli*, *Arabidopsis* and human enzymes which are shown in Fig. 5 ▶ corroborate this suggestion and demonstrate that the rigid body formed of domains 2 and 3 of the *B. megaterium* enzyme has a very similar orientation to that of the *E. coli* enzyme, with which it shares 48% sequence identity. Indeed, the domain shifts of the *B. megaterium* and *E. coli* enzymes seem to lie between the larger movements exhibited by the *Arabidopsis* and human PBGDs (Fig. 5 ▶), which have slightly lower sequence identities of 43 and 47% to the *B. megaterium* enzyme, respectively.

### 3.2. Definition of the cofactor redox states   

**Figure 6:**
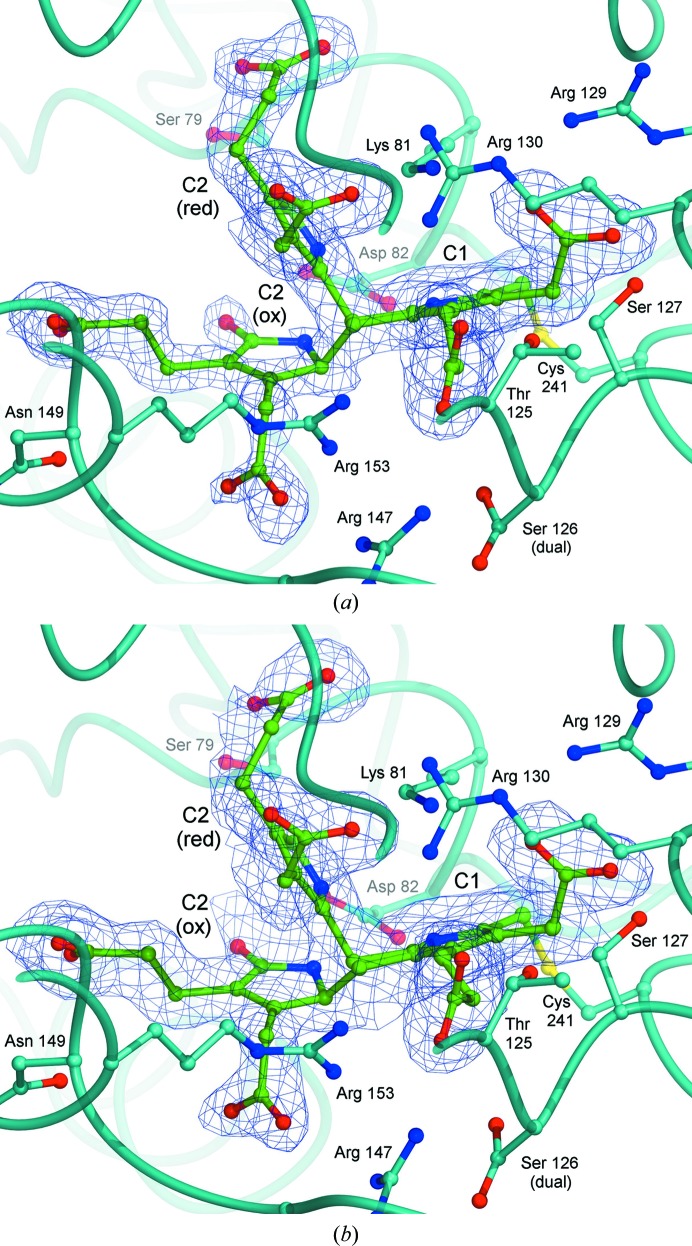
Electron density showing the dual conformations of the cofactor. The local fold of the protein is indicated as a cyan tube and the side chains adjacent to the cofactor are shown in the same colour as the enzyme, while the cofactor itself is coloured green. The map obtained for the 40-­day-old protein is shown in (*a*), with that for the 50-day-old protein shown in (*b*); both maps are contoured at 0.7 r.m.s.. The C2 ring clearly adopts two positions depending on its oxidation state, which are shown as C2(ox) and C2(red). In the oxidized conformation, the C2 ring possesses a carbonyl O atom substituted at the α-position; accordingly, the proportion of cofactor adopting this conformer increases with time.

In the electron-density maps for both structures presented here, the dipyrromethane cofactor, which is covalently attached to Cys241, is very well defined (Fig. 6 ▶). The two rings of the cofactor (labelled C1 and C2) are held within a large highly conserved cleft between domains 1 and 2 of the protein. The cleft has a preponderance of basic residues, most notably arginine, which interact electrostatically with the carboxylate side groups of the pyrroles. At the base of the cleft, an invariant aspartic acid, Asp82 in *B. megaterium* PBGD, forms hydrogen bonds to both the C1 and C2 pyrrole-ring N atoms. These interactions have been described in great detail elsewhere for high-resolution structures of related enzymes (*e.g.* Louie *et al.*, 1992 ▶, 1996 ▶; Roberts *et al.*, 2013 ▶), and the high degree of conservation of these critical amino acids (see Fig. 4 ▶) means that essentially the same interactions are observed in the *B. megaterium* structure reported here. However, one interesting difference is that the flap covering the active site of *B. megaterium* PBGD (residues 42–62) completely lacks electron density, despite many efforts to rebuild it during refinement of the two structures reported here. This suggests that the active-site flap of *B. megaterium* PBGD is rather more disordered than in the structures of other related enzymes. It is not immediately obvious why this should be the case since the flap sequence is quite highly conserved in PBGD enzymes (Fig. 4 ▶), although one factor may be the complete absence of crystal contacts in this region of the *B. megaterium* structure. In principle, the greater flexibility of the flap may confer the cofactor of the *B. megaterium* enzyme with greater flexibility and sensitivity to environmental factors such as pH, ionic strength and redox state. Indeed, reducing SDS–PAGE and mass spectrometry confirmed that cleavage of the enzyme occurs over a period of several days during storage on ice, giving a reduction in its molecular mass from 35 to 28 kDa. This is consistent with cleavage by trace proteases somewhere within the highly disordered active-site flap region between residues 42 and 62 during storage. It may be interesting that kinetic studies of the enzyme showed that whilst it has a *k*_cat_ that is comparable to those of other PBGDs (0.1 s^−1^), its *K*_m_ of 100 µ*M* is rather high for this enzyme family (Jordan, 1991 ▶), although it is comparable to the values found for algal PBGDs (Battersby *et al.*, 1983 ▶). Hence, it is conceivable that a lower affinity for substrate may originate from greater disorder in the active-site flap region of the enzyme.

**Figure 7:**
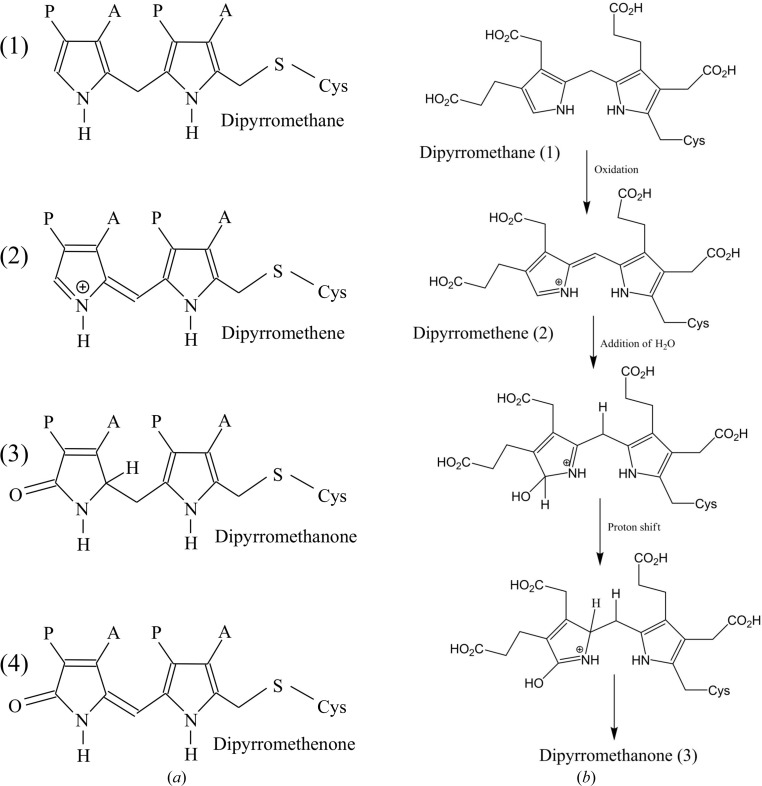
Oxidation states of the dipyrromethane cofactor. Different possible oxidation states of the dipyrromethane cofactor are shown in (*a*) along with a proposed mechanism for oxidation of the dipyrromethane to dipyrromethene and subsequently dipyrromethanone (*b*).

Another interesting feature of *B. megaterium* PBGD is the pink colouration which is found in the cells expressing the enzyme and in the final affinity-purified enzyme (Azim *et al.*, 2013 ▶). Over time the protein solution becomes progressively yellow and the crystals we obtained were indeed of this colour, as is the case for most PBGDs studied to date. Prior to structure analysis of the *B. megaterium* enzyme, we anticipated that the initial pink coloration might indicate the presence of a previously unseen polypyrrole intermediate or an unusual oxidation product of the dipyrromethane cofactor. Structural studies of the *E. coli* and *A. thaliana* PBGD enzymes have shown that the cofactor becomes oxidized to the predominantly planar dipyrromethenone form, in which the α-position of the terminal pyrrole ring (ring C2) is oxidized, as shown in Fig. 7 ▶. This form of the enzyme is catalytically inactive owing to the usual attachment site for incoming pyrroles being blocked by a carbonyl O atom: an effect that probably accounts for the slow inactivation of the enzyme over 2–3 weeks during storage or crystallization.

In both of the structures of *B. megaterium* PBGD that we report here, the C2 ring clearly adopts two positions (Fig. 6 ▶), one of which corresponds to the fully reduced dipyrromethane that has been observed in structures of the *E. coli* and human enzymes when they are crystallized under reducing conditions (Hädener *et al.*, 1999 ▶; Song *et al.*, 2009 ▶). The other position of the C2 ring corresponds approximately to that of the oxidized cofactor in the high-resolution structures of the *E. coli* and *Arabidopsis* enzymes when the crystals were grown under oxidizing conditions (Louie *et al.*, 1996 ▶; Roberts *et al.*, 2013 ▶). In these structures, the C2 ring of the cofactor has a carbonyl oxygen substituent at the α-position (see Fig. 7 ▶ *a*), which will render the enzyme inactive (see above). In the dipyrromethenone state, both rings of the cofactor are found to be approximately coplanar and, although they are not constrained to be so by the molecular geometry, this effect may facilitate the delocalization of electrons over both pyrrole rings. In the *B. megaterium* enzyme the electron-density map clearly shows that the oxidized conformation of the cofactor has the same oxygen substituent in the C2 ring, which is suggestive of the same dipyromethenone form. However, the other α-position of the C2 ring which partakes in the methylene bridge with the adjacent C1 pyrrole is clearly tetrahedral, in contrast to the trigonal planar configuration that is found in dipyrromethenone. This suggests that in the *B. megaterium* PBGD structure the ‘oxidized’ conformation of the cofactor is actually the partly reduced dipyrrometh**a**none form, the formula for which is shown in Fig. 7 ▶(*a*). Accordingly, refinement of the occupancies of the oxidized and reduced states in the two structures reported here showed that the proportion of the oxidized dipyrromethanone form increases in a time-dependent manner from 30% in the case of the 40-day-old protein to 50% for the 50-­day-old protein.

As mentioned above, the intriguing pink colouration of the freshly purified protein gradually changes to yellow over a 2–­3-week period. One possibility is that the pink colour is owing to the dipyrromethene state (Fig. 7 ▶), which is derived from the fully reduced cofactor dipyrromethane by partial oxidation. Since the protonated dipyrromethene has a positive charge delocalized over two conjugated rings, one therefore might expect it to be more intensely coloured than the other intermediates shown in Fig. 7 ▶. In an electron-density map, a dipyrromethene would be expected to look exactly like the fully reduced dipyrromethane because it only differs from the latter by the loss of an H atom from the bridging C atom. The yellow colour that accumulates over time could therefore be owing to the dipyrromethanone (as shown in Fig. 7 ▶), which arises from further oxidation of the dipyrromethene. A proposed mechanism for this process is shown in Fig. 7 ▶(*b*).

To summarize, the native enzyme is most likely to contain a dipyrromethane, which oxidizes to a dipyrromethene with an extended chromophore giving the red-shifted spectrum. In a time-dependent fashion, the dipyrromethene is converted into the yellow dipyrromethanone form. The latter conversion, as shown in Fig. 7 ▶(*b*), merely requires the addition of water to the protonated Schiff base followed by prototropic rearrangements. According to this mechanism, the dipyrromethenone is the further oxidation product of the dipyrromethanone.

The two structures reported here superimpose very closely, with an r.m.s.d. of only 0.17 Å for all C^α^ atoms. Since analysis of the *A. thaliana* PBGD structure suggested that domains 2 and 3 appeared to move slightly relative to domain 1 of the enzyme as a rigid unit (Roberts *et al.*, 2013 ▶), the same analysis was conducted for both of the *B. megaterium* structures. Interestingly, domains 2 and 3 appear to rotate towards domain 1 by approximately 0.6° in the more oxidized structure, suggesting that as the proportion of cofactor in the more extended state increases, the active site of the enzyme is able to close more. This rigid-body rotation gives rise to movements of several loops over the active site of at most 0.5 Å. Whilst this domain movement would appear to be consistent with the more extended conformation of the oxidized cofactor, allowing the active-site cleft to close, it should be noted that the scale of the loop movements is of the order of the differences in unit-cell parameters. Thus, whether these domain movements are a cause or an effect of the slightly different crystal lattice parameters cannot easily be determined.

## 4. Discussion   

Porphobilinogen deaminase is an intriguing enzyme and few details of how it catalyses the tetrapolymerization of pyrroles are well understood, although a number of possible models for the elongation process have been suggested (Jordan, 1991 ▶; Louie *et al.*, 1992 ▶). One possibility is that domain movements cause the bound cofactor and polypyrrole intermediates to move past the catalytic centre of the enzyme in a stepwise manner, thus permitting the binding of additional substrate moieties and the completion of the tetrapyrrole product. This model would allow the condensation reactions to be driven by the extensive interactions that we observe between the enzyme and the cofactor, coupled with acid–base catalysis provided by the invariant aspartate residue (Asp82 in the PBGD from *B. megaterium*). Whilst the reported structure confirms all of these features, it is unusual in that the enzyme has a highly disordered flap over the active site, with some 20 amino-acid residues being invisible in the electron density. One consequence of this is that the full extent of the cavities around the active-site crevice becomes apparent and hints at the numerous possibilities for accommodating the polypyrrole intermediates of the elongation reaction. Another model of the elongation reaction is that the cofactor remains close to the positions observed in the X-ray structure as an incoming pyrrole is condensed with it. If the newly added pyrrole can then be repositioned, this local movement may be sufficient to ‘free-up’ the catalytic apparatus for the next elongation cycle. In the reduced conformation, the C2 ring of the cofactor is buried further within the active-site cavity, which creates more space for an incoming pyrrole to bind in the vicinity of the catalytic aspartate without displacement of the cofactor. However, the tight geometric constraints of the active site are such that steric hindrance between the cofactor and an incoming pyrrole would probably force the C2 ring into a conformation in which its free α-position would be even less accessible to the incoming substrate. Regardless of the mechanism of elongation, movement of the cofactor is most likely to be linked to movement of the protein domains, and the current structure provides further corroboration for a model in which domains 2 and 3 of the protein appear to move relative to domain 1 as a fairly rigid unit (Roberts *et al.*, 2013 ▶). In this respect, it is interesting that domains 2 and 3 provide the bulk of the binding interactions with the cofactor, whilst domain 1 provides the main catalytic residue of the enzyme, Asp82.

PBGD enzymes are highly sensitive to oxidation of the dipyrromethane cofactor, which causes the protein to become notably yellow with time (Jordan, 1991 ▶; Louie *et al.*, 1996 ▶). Intriguingly, the *B. megaterium* enzyme has a pronounced pink colour during overexpression and purification (Azim *et al.*, 2103), although the purified protein slowly becomes yellow over a period of a few weeks. Our structural analyses of the *B. megaterium* enzyme at two different time points after purification establish the different oxidation states of the cofactor that are likely to be responsible for these effects. The native enzyme will only be active with fully reduced dipyrromethane at the active site. However, the more exposed nature of the active-site cleft in this enzyme when compared with other PBGD structures suggests that the cofactor may be particularly prone to oxidation to the dipyrromethene form, which is highly conjugated and is likely to have a red-shifted spectrum when protonated. Protonation of the dipyrromethene gives it a positive charge on one of the pyrrole N atoms which can readily be stabilized by its close proximity to the catalytic aspartate Asp82. Whilst discrimination between dipyrromethene and dipyrromethane would not be possible by X-ray diffraction analysis at the resolutions reported here, there are compelling reasons to believe that the pink colouration is owing to the presence of the partly oxidized protonated dipyrromethene form. Addition of water to this dipyrrin converts it to the yellow dipyrromethanone form, which possesses a tetrahedral α-C atom in the terminal pyrrole. The time-dependent accumulation of this intermediate is confirmed by our X-ray analyses. Further oxidation of the dipyrromethanone will ultimately lead to the dipyrromethenone form, which has been confirmed in other high-resolution PBGD structures (Louie *et al.*, 1996 ▶; Roberts *et al.*, 2013 ▶). The loss of catalytic activity as the cofactor is oxidized to the dipyrromethanone form cannot be reversed by the addition of mild reducing agents. Indeed, there is no known reducing agent that will reverse the oxidized cofactor to its original redox state in a predictable fashion and still maintain the integrity of the protein structure.

## Supplementary Material

PDB reference: porphobilinogen deaminase, 4mlv

PDB reference: 4mlq

## Figures and Tables

**Table 1:** Data-collection and processing statistics for *B. megaterium* porphobilinogen deaminase Values in parentheses are for the outer resolution shell.

Data set	Crystal 1	Crystal 2
Beamline	I03, DLS	BM14, ESRF
Wavelength (Å)	0.976	1.072
Space group	*P*2_1_2_1_2_1_	*P*2_1_2_1_2_1_
Unit-cell parameters (Å)		
*a*	53.32	53.01
*b*	65.78	65.12
*c*	97.21	96.78
Mosaic spread (°)	0.26	1.01
Resolution (Å)	48.60–1.46 (1.53–1.46)	31.33–1.60 (1.69–1.60)
*R*_merge_ † (%)	6.1 (55.9)	8.8 (149.3)
CC_1/2_ ‡ (%)	99.8 (86.7)	99.8 (33.1)
*R*_meas_ § (%)	6.7 (61.3)	9.5 (168.4)
Completeness (%)	100.0 (100.0)	95.6 (88.7)
Average *I*/σ(*I*)	14.4 (3.0)	10.1 (0.9)
Multiplicity	6.2 (6.0)	6.4 (4.5)
No. of observed reflections	378329 (52575)	273058 (25347)
No. of unique reflections	60772 (8748)	42866 (5649)
Wilson plot *B* factor (Å^2^)	15.8	17.0
*R* factor (%)	14.1	16.1
Free *R* factor (%)	18.6	24.2
R.m.s.d., bond lengths (Å)	0.024	0.019
R.m.s.d., bond angles (°)	2.44	2.14
No. of reflections in working set	57626	40552
Mean protein *B* factor (Å^2^)	24.6	28.2

† *R*_merge_ = 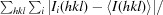 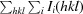.

‡ CC_1/2_ is the half-set correlation coefficient as described by Karplus & Diederichs (2012 ▶).

§ *R*_meas_ = 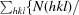 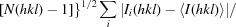 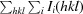, where 〈*I*(*hkl*)〉 is the mean intensity of the *N*(*hkl*) observations *I_i_*(*hkl*) of each unique reflection *hkl* after scaling.
